# The perplexity of targeting genetic alterations in hepatocellular carcinoma

**DOI:** 10.1007/s12032-020-01392-8

**Published:** 2020-07-22

**Authors:** Michele Barone, Alfredo Di Leo, Carlo Sabbà, Antonio Mazzocca

**Affiliations:** 1 Gastroenterology Unit, Department of Emergency and Organ Transplantation (D.E.T.O.), University of Bari School of Medicine, Piazza G. Cesare, 11, 70124 Bari, Italy; 2 Interdisciplinary Department of Medicine, University of Bari School of Medicine, Piazza G. Cesare, 11, 70124 Bari, Italy

**Keywords:** Hepatocellular carcinoma, Genetic alterations, Tumor heterogeneity, Gene-targeted therapy, Precision medicine, Integrative approach

## Abstract

Genetic heterogeneity is a well-recognized feature of hepatocellular carcinoma (HCC). The coexistence of multiple genetic alterations in the same HCC nodule contributes to explain why gene-targeted therapy has largely failed. Targeting of early genetic alterations could theoretically be a more effective therapeutic strategy preventing HCC. However, the failure of most targeted therapies has raised much perplexity regarding the role of genetic alterations in driving cancer as the main paradigm. Here, we discuss the methodological and conceptual limitations of targeting genetic alterations and their products that may explain the limited success of the novel mechanism-based drugs in the treatment of HCC. In light of these limitations and despite the era of the so-called “precision medicine,” prevention and early diagnosis of conditions predisposing to HCC remain the gold standard approach to prevent the development of this type of cancer. Finally, a paradigm shift to a more systemic approach to cancer is required to find optimal therapeutic solutions to treat this disease.

## Introduction

In the last decade, numerous studies have provided several and accurate details on the genetic alterations associated with hepatocellular carcinoma (HCC). In particular, the familial genetic alterations responsible for the development of cirrhosis and associated with HCC formation have been entirely unraveled [1]. Moreover, many efforts have been made to recognize the genetic alterations that are observed in the tumor tissue, since they could be used as potential theranostic targets or could improve decisions/therapies in HCC [1, 2]. However, despite the considerable efforts made in this field and a large number of identified genes (Table 1), the presence of tumor genetic heterogeneity among patients as well as the coexistence of different types of genetic alterations in the same nodule still represent two unsolved problems that explain the limited success of the novel mechanism-based drugs in the treatment of HCC.

**Table 1:** Prevalence of main genetic alterations involved in hepatocarcinogenesis

Gene	Type of genetic alteration	Function	Prevalence	Reference (year)
TERT (telomerase reverse-transcriptase)	Mutation (nucleotide substitutions) HBV-DNA insertions	Increased telomerase expression	60–90 (%)	Nault (2013) [3] Bruix (2015) [4] Nault (2015) [5]
CTNNB1 (cadherin-associated protein β1)	Exon 3 deletion Missense mutations HBV-DNA insertions	Activation of β-catenin Associated with IL6/JAK/STAT activation and inflammation	25–62 (%)	Huang (2012) [6] Guichard (2012) [7] Kan (2013) [8] Tian (2015) [9] Nault (2017) [10]
TP53 (tumor Protein 53)	Mutation codon 249	Loss of function as tumor suppressor gene Gain of function as oncogene Loss of regulation of the immune response	13–48 (%)	Guichard (2012) [7] Takai (2014) [11] Schulze (2015) [12] Yamamoto (2018) [13] Long (2019) [14]
CDKN2A (cyclin-dependent kinase inhibitor 2A)	Homozygous deletions/mutations or epigenetic silencing	Loss of function as tumor suppressor gene	2–12	Guichard (2012) [7] Totoki (2014) [15] Schulze (2015) [16] Tian (2015) [9]
VEGFA (vascular endothelial growth factor A)	Gene amplification	Promotion of angiogenesis Stimulation of HGF production	7–11%	Zucman (2015) [1] Chiang ‘(2008) [17] Llovelet (2016) [18] Oh (2019) [19] Horwitz (2014) [20]
FGF19 (fibroblast growth factor 19)	Gene amplification	Proliferative signaling Anti-apoptosis	6.5%	Raja (2019) [21] [22]
AXIN1-2, ARID2, ARID1A, TSC1/TSC2, KEAP1, MLL2 RPS6KA3	Multiple mechanisms	WNT/β-catenin pathway SWI/SNF chromatin remodeling complexes Activation of the AKT/MTOR signaling Control of histone methylation	Low frequency	Zucman (2015) [1] Nault (2017) [10]

As shown in Table 1, a variety of genetic alterations have been reported in HCC. Mutations due to nucleotide substitutions or HBV-DNA insertions leading to an increased telomerase reverse-transcriptase (TERT) gene expression in HCC, with a prevalence of 60–90%, have been reported [3–5]. Deletion of exon three, missense mutations, and HBV-DNA insertions in the Cadherin-associated protein β1 (CTNNB1) gene, causing β-catenin and IL6/JAK/STAT activation and the inflammatory response sustenance have been also reported [6–10]. Mutation at codon 249 of the Tumor Protein 53 (TP53) gene has been described in 13–48% of HCC [7–14]. Furthermore, homozygous deletions/mutations or epigenetic silencing of cyclin-dependent kinase inhibitor 2A (CDKN2A) have been shown to contribute to the loss of function of this tumor suppressor gene in HCC [7, 9, 15, 16]. Finally, amplification of vascular endothelial growth factor A (VEGFA) [1, 17–20] and Fibroblast growth factor 19 (FGF19) gene [21, 22] predisposing to angiogenic and pro-proliferative signaling were found in 7–11% and 6.5% of HCC, respectively.

Based on these considerations, we can assert that the pharmacological principles of potential therapeutic agents targeting genetic alterations have been probably based on the wrong paradigm [23]. First, the presence of multiple genetic alterations itself represents an a priori mechanism of resistance and, theoretically, a barrier hard to overpass in terms of drug design and therapeutic strategy. Second, targeting a single gene or mutation often results in relapse since tumor cells easily escape or compensate for this type of block. Third, it is hard to establish which mutation or alteration is the most relevant to cancer formation or progression in a determined type or subset of HCC. Fourth, the presence of a genetic alteration could not necessarily be considered the driver of cancer promotion but could, instead, represent the consequence of uncontrolled proliferation. Fifth, numerous oncogenic alterations are currently undruggable. Several drugs against genetic alterations or their products have proven ineffective in terms of benefits on overall survival and quality of life in different forms of cancer [24].

The failure of these therapeutic strategies reinforces the importance of conventional therapies (liver transplantation, surgical resection, locoregional therapies), which used alone or in combination, remain the only widely accepted clinical treatments for HCC [25–27]. Therefore, the recognition of the subjects at risk, essentially cirrhotics, strictly monitored mainly by echotomography and suitable serum markers (α-fetoprotein), and the initiation of early treatment remain the commonly used strategy to cure, even if it may be considered suboptimal in the era of “precision medicine” [28]. However, in the last years, the publication of a large number of experimental and clinical studies on the possible role of immunotherapy to cure HCC could represent a new promising perspective [29].

An entirely different approach to the cure of HCC could consist of the prevention of the disease through the identification and targeting of the early genetic alterations that anticipate the neoplastic transformation. Unfortunately, this strategy seems also not to succeed in the difficulty to target/correct familial genetic alteration and oncogenic mutations. In this contest, the HBV transgenic mouse model offers the opportunity to study the process of tumor initiation and progression related to the HBV infection [30, 31], and to develop the targeting of the early genetic alterations. Using this model, an early, pre-neoplastic up-regulation of two genes namely cyclin-dependent kinase inhibitor 2D (Cdkn2d) and stromal cell-derived one alpha receptor (CXCR4) was detected [30]. Cdkn2d is expressed in HCC nodules and stimulates cell proliferation, whereas CXCR4 is associated with an immunosuppressive tumor microenvironment [9, 30, 32]. Moreover, using a similar HBV transgenic mouse model, Sun et al. [31] uncovered a set of genes up-regulated in different phases of hepatocarcinogenesis including Trefoil Factor 3 (TFF3), Insulin-like growth factor 2 (IGF2), Lipoprotein Lipase (LPL), and Secreted Phosphoprotein 1 (SPP1, the homolog of human Osteopontin). Finally, Nault et al. found a bi-allelic inactivation of TCF1 in hepatic adenomas, which is related to inactivating mutations of HNF1A [10]. However, further studies are needed to confirm if what found in animal models is translatable to humans.

Another important aspect regarding the development of new potential therapeutic agents against HCC that should be taken into account is the role of metabolic-induced modifications acting as environmental modifiers with the ability to influence a susceptible genetic or epigenetic background [33]. In this context, particular importance is assuming the emerging non-alcoholic fatty liver disease (NAFLD), an association of microenvironmental inflammation, aberrant metabolism, and liver regeneration [33] that could confer an increased risk of HCC, regardless of cirrhosis [34]. Current anti-HCC pharmacological approaches are still unsatisfactory and mainly rely on tyrosine-kinase inhibitors, such as sorafenib [35, 36]. Alpha-fetoprotein (AFP) remains the commonly used diagnostic biomarker since promising markers quite often not directly correlate with the tumor burden [37]. Also, the advanced phase of the neoplastic disease still faces pharmacological challenges [38]. We have been working on identifying novel pharmacological targets and therapeutic strategies in HCC [39–42]. However, there is still a long way to go in the field of anti-HCC drug strategies. Also, the prevalent paradigm mainly based on the gene-centered theories could not help design a more integrative pharmacology. A systemic approach to cancer may instead be significant to a broad therapeutic approach [23, 43–45]. Therefore, HCC prevention or treatment of metabolic conditions including NAFLD still represents an important integrative approach that may probably be more achievable and effective to prevent HCC development and progression, according to the principle of the complexity of systems biology including cancer biology.
